# Profunda Femoris Artery Pseudoaneurysm After Proximal Nailing: A Case Report

**DOI:** 10.7759/cureus.100899

**Published:** 2026-01-06

**Authors:** Seyyid Y Maatou, Simon Koulischer, Denis Bataille, Bartholome Laplantine

**Affiliations:** 1 Orthopaedics and Traumatology, CHU Saint-Pierre Site Porte de Hal, Brussels, BEL; 2 Vascular Surgery, CHU Saint-Pierre Site Porte de Hal, Brussels, BEL

**Keywords:** case report, ct angiography, femoral fracture, intramedullary fixation, profunda femoris artery, pseudoaneurysm, vascular complication

## Abstract

Hip fractures in elderly patients are common after low-energy trauma, while vascular complications remain rare but potentially life-threatening. A pseudoaneurysm of the profunda femoris artery may occur when displaced bone fragments come into close contact with the vessel. We report the case of a 68-year-old man who sustained a displaced subtrochanteric fracture treated with gamma nail fixation. The initial postoperative course was uneventful; however, a secondary fall on postoperative day seven led to implant displacement, progressive anemia, and thigh swelling. Computed tomography angiography revealed a large profunda femoris artery pseudoaneurysm in direct contact with a displaced lesser trochanter fragment, confirming the causal role of this fragment. Management required a combined orthopedic and vascular approach, including fragment reduction and fixation, followed by endovascular stent placement. At the two-month follow-up, fracture union was achieved, and the patient regained pain-free mobility with crutches. This case highlights that vascular injury should be suspected in patients with persistent anemia or swelling after hip fracture fixation, particularly following secondary trauma, and emphasizes the importance of early vascular imaging and multidisciplinary management to prevent severe complications.

## Introduction

A pseudoaneurysm is a contained hematoma resulting from partial disruption of the arterial wall. Unlike a true aneurysm, it lies outside the arterial layers and communicates with the parent vessel through a narrow neck, forming a perfused sac. It most commonly occurs after trauma, arterial puncture during interventional procedures, or infection [1]. Pseudoaneurysm of the profunda femoris artery (PFA) is an exceptionally rare but potentially life-threatening complication following fixation of pertrochanteric or subtrochanteric femoral fractures [2,3]. Only a limited number of cases have been reported in the literature. Its rarity, combined with nonspecific clinical manifestations such as thigh swelling, progressive anemia, or pain, frequently leads to delayed diagnosis. The condition may be misinterpreted as a postoperative hematoma, infection, or deep vein thrombosis. If left untreated, however, a PFA pseudoaneurysm carries a significant risk of rupture, severe hemorrhage, and distal ischemia [4,5]. Anatomically, the PFA courses are in proximity to the femoral shaft in the subtrochanteric and pertrochanteric regions, rendering it vulnerable during fracture fixation. Vascular injury may result from drill trajectory, screw penetration, or repeated contact with sharp bone fragments, particularly the displaced lesser trochanter. Advanced age-related vascular fragility, exacerbated by atherosclerosis, muscle atrophy, or anticoagulant therapy, further increases susceptibility [6]. Chronic mechanical irritation by a displaced fragment can cause progressive erosion of the arterial wall, explaining the delayed onset of pseudoaneurysm formation, sometimes days or weeks after surgery. Clinically, persistent anemia or progressive thigh swelling should prompt vascular evaluation. While Doppler ultrasound may serve as an initial screening tool, CT angiography is the diagnostic modality of choice, as it accurately delineates the vascular lesion and its relationship to fracture fragments and implants. We report the case of a 68-year-old patient with a pertrochanteric femoral fracture treated with a gamma nail who developed a large PFA pseudoaneurysm only after a second traumatic event, associated with secondary displacement of the lesser trochanter fragment. To our knowledge, this delayed presentation following secondary trauma, with clear radiologic and intraoperative confirmation of causality, is uncommon. This case highlights the importance of early clinical suspicion, appropriate vascular imaging, and coordinated orthopedic-vascular management. This case report is presented in accordance with the Surgical CAse REport (SCARE) checklist.

## Case presentation

A 68-year-old man with stage IVa lung adenocarcinoma presented after a fall at home. He complained of pain and inability to bear weight on the left lower limb. His functional status before the fracture was excellent (Parker Mobility Score 9/9). He had no vascular risk factors or significant comorbidities.

**Figure 1:**
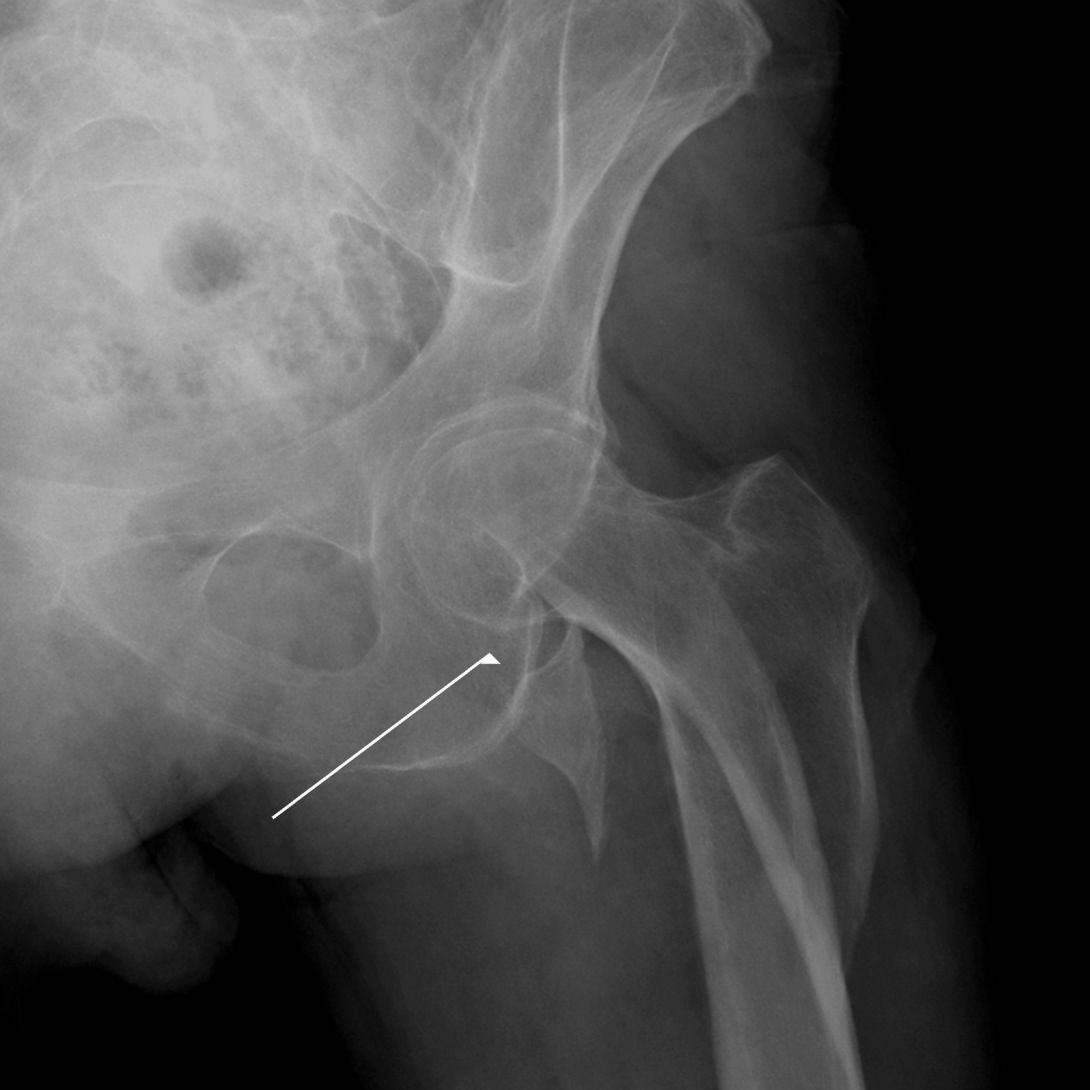
Subtrochanteric fracture with avulsion of the lesser trochanter, significant diastasis estimated at 2.5 cm.

Radiographs revealed a displaced subtrochanteric femoral fracture (AO/OTA 31A3.3) with avulsion of the lesser trochanter and minor greater trochanter involvement (Figure 1). There were no radiographic features suggestive of a pathological fracture, and the fracture was considered traumatic.

**Figure 2:**
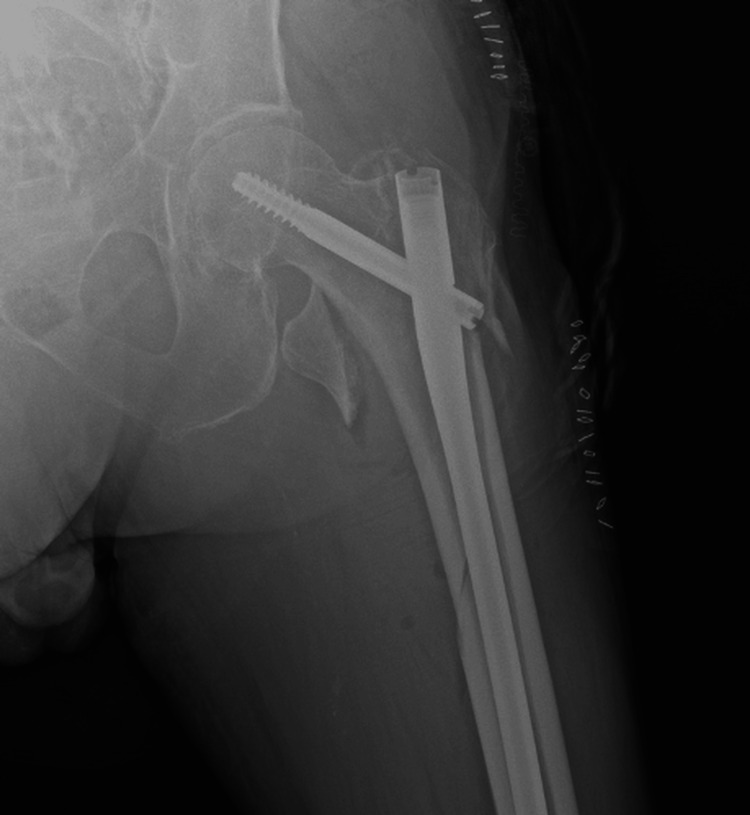
Postoperative X-ray Showing Intramedullary Gamma Nail Fixation of the left Subtrochanteric Femoral Fracture.

He underwent fixation with a short gamma nail (Figure 2). The immediate postoperative course was uneventful, and rehabilitation was started.

On postoperative day (POD) 7, he sustained a second fall. He subsequently presented with progressive thigh swelling and external bleeding, recurrent pain, and pallor. On clinical examination, he was hemodynamically stable, with intact distal pulses, no sensory or motor deficit, and no clinical signs of compartment syndrome; no pulsatile mass or bruit was detected. Laboratory investigations revealed a marked drop in hemoglobin (from 9 g/dL to 5.9 g/dL) associated with an elevated C-reactive protein level (122 mg/L). Despite blood transfusion, anemia persisted, raising suspicion of ongoing occult bleeding and prompting further vascular evaluation (Table 1).

**Table 1:** Chronological evolution of hematological and inflammatory parameters, transfusion requirements, and anticoagulation status

Parameter	13/05/2025 (POD 5)	20/05/2025 (POD 11)	22/05/2025 (POD 13)	Reference range
WBC (/mm³)	9.8	9.3	8.6	4.0–10.0
Neutrophils (%)	81.6	83.0	83.2	40–75
Hemoglobin (g/dL)	9.0	5.9	6.9	13.0–17.0
CRP (mg/L)	45.3	98.0	122.0	<5.0
ESR (mm/h)	—	—	50	<20
Ferritin (ng/mL)	—	—	826	30–400
Blood transfusion	1-unit PRBC	2 units PRBC	1-unit PRBC	—
Anticoagulation/VTE prophylaxis	Low-Molecular-Weight Heparin (LMWH), prophylactic dose	LMWH, prophylactic dose	LMWH, prophylactic dose	—

**Figure 3:**
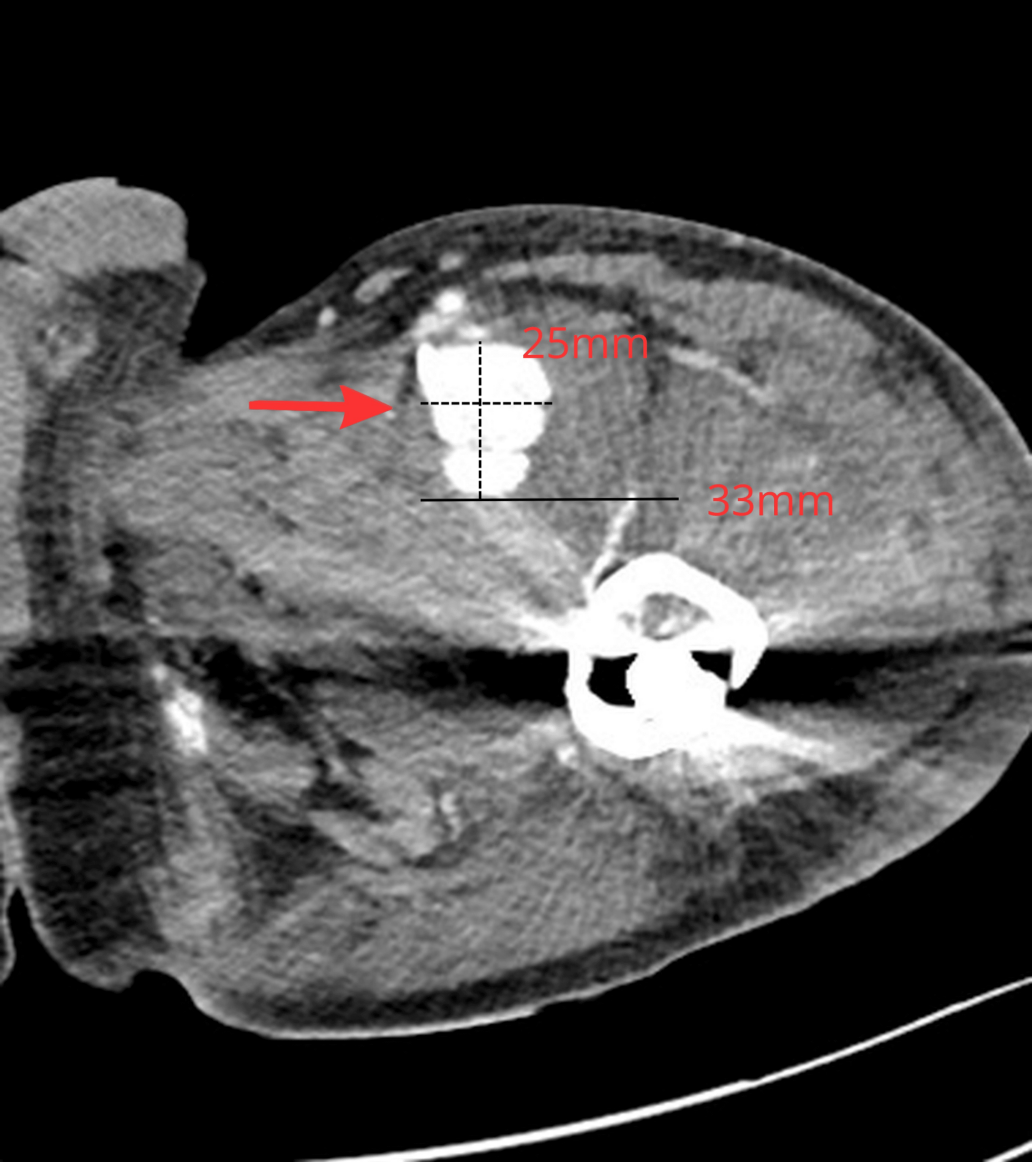
CT angiography demonstrating a pseudoaneurysm of the left PFA. No vascular abnormality was present on a PET-CT scan performed 3 weeks prior to the traumatic event. The pseudoaneurysm measures approximately 25 mm in maximal diameter and is in direct spatial contact with the displaced lesser trochanter fragment, with a measured distance of approximately 33 mm.

**Figure 4:**
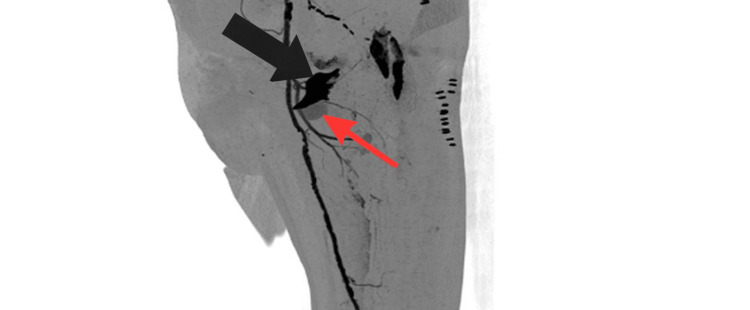
CT angiography of the left lower limb showing a large pseudoaneurysm of the profunda femoris artery. The black arrow points to the displaced lesser trochanter fragment, and the red arrow identifies the associated pseudoaneurysm of the profunda femoris artery.

CT angiography and Digital Subtraction Angiography (DSA) demonstrated a large pseudoaneurysm of the PFA (Figures 3-4).

**Figure 5:**
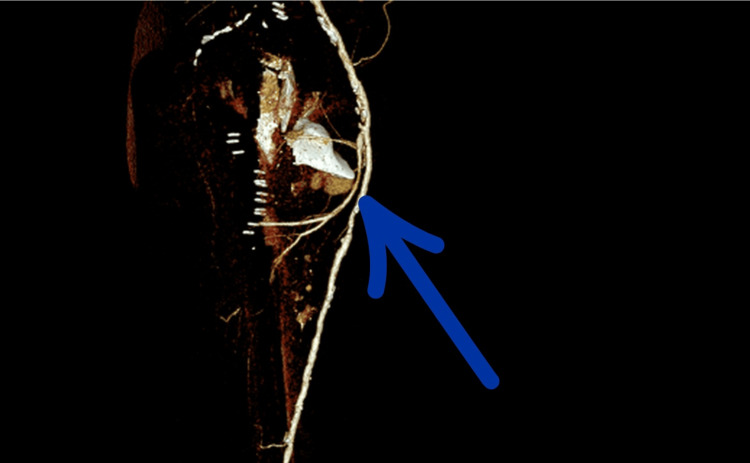
CT angiography of the left lower limb showing a large pseudoaneurysm of the profunda femoris artery, in close anatomical proximity to the displaced lesser trochanter fragment (right side view). Direct contact between the displaced lesser trochanter fragment and the profunda femoris artery is clearly visible.

**Figure 6:**
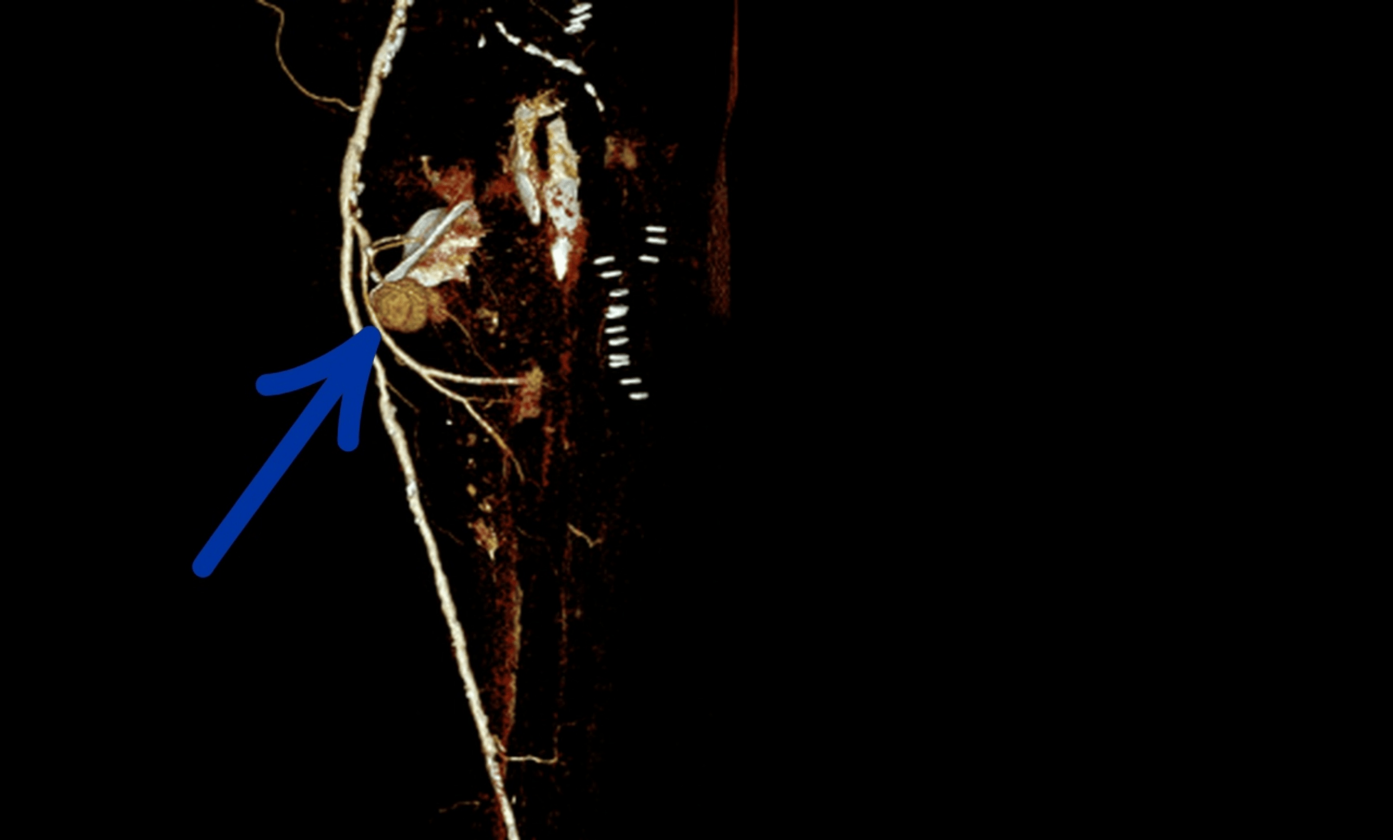
CT angiography of the left lower limb illustrating the pseudoaneurysm of the profunda femoris artery, in direct contact with the free bony fragment after secondary displacement (left side view). Direct contact between the displaced lesser trochanter fragment and the profunda femoris artery is clearly visible.

Comparison with earlier postoperative radiographs confirmed secondary fragment displacement, supporting a likely mechanical contribution to arterial injury. CT angiography demonstrated direct contact between the displaced lesser trochanter fragment and the PFA at the level of the pseudoaneurysm, with loss of the normal intervening soft-tissue plane (Figures 5-6).

**Figure 7:**
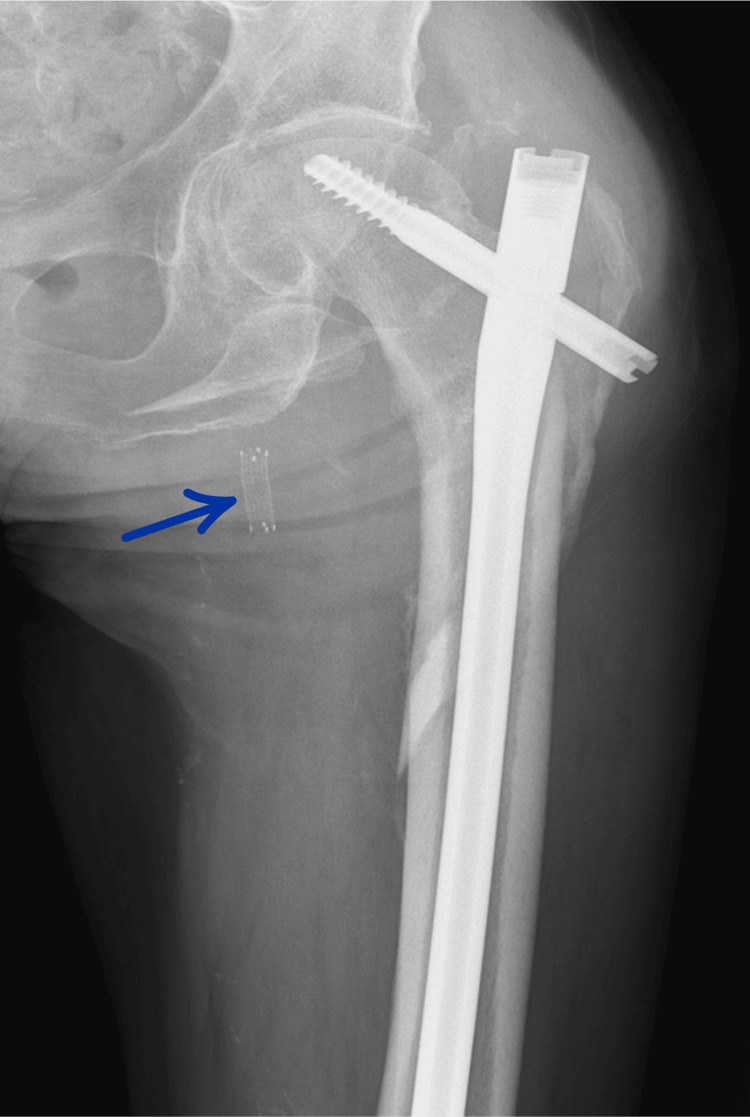
Three-week postoperative radiograph showing the vascular stent in place and early signs of fracture consolidation

**Figure 8:**
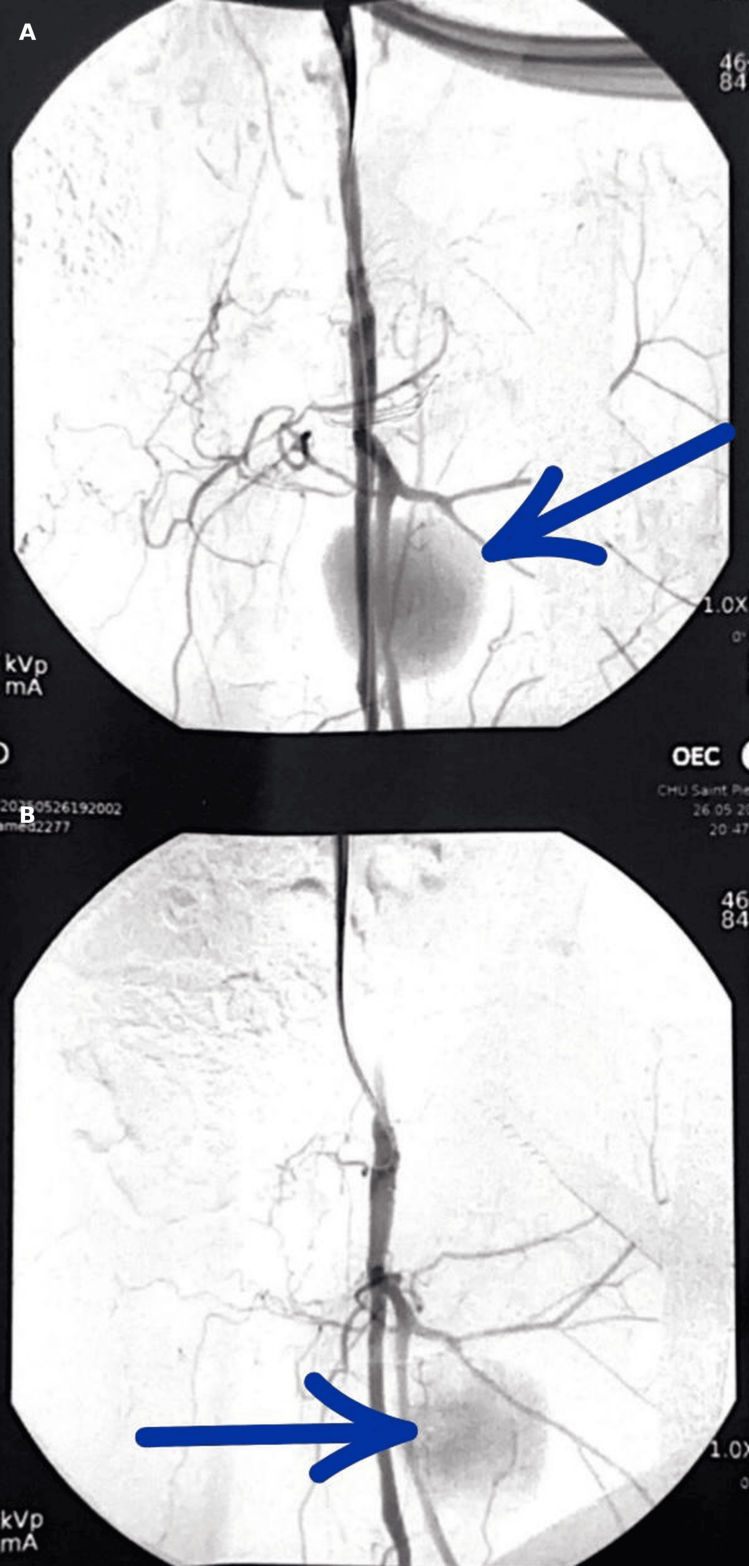
Digital subtraction angiography demonstrating a large profunda femoris artery pseudoaneurysm before endovascular treatment. (A) Anteroposterior projection demonstrating a contrast-filled pseudoaneurysm (arrow). (B) Oblique projection of the same pseudoaneurysm (arrow).

**Figure 9:**
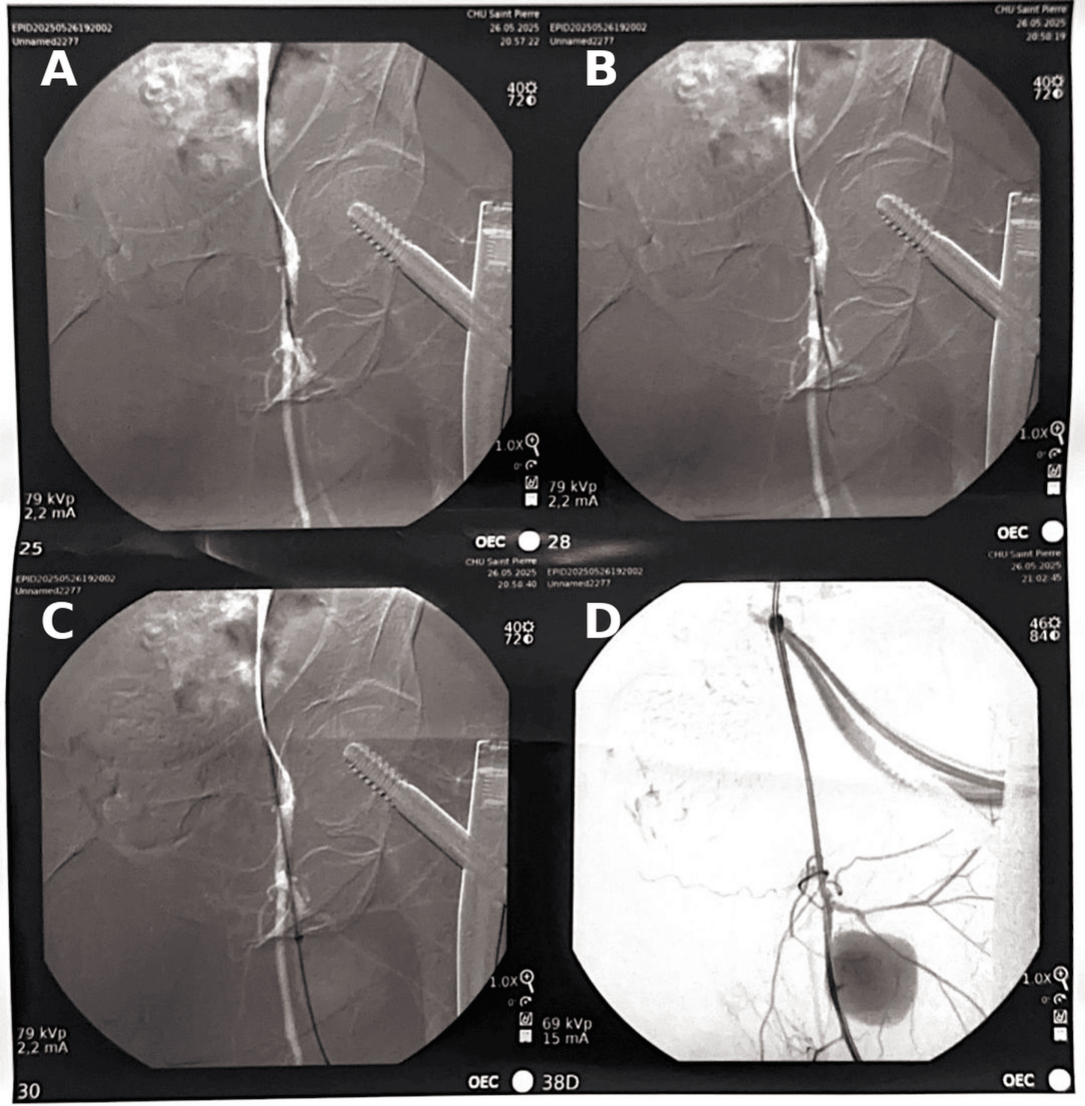
Intraoperative angiography showing selective catheterization of the profunda femoris artery and deployment of a covered stent across the pseudoaneurysm neck. (A) Initial angiography demonstrating the pseudoaneurysm and its neck arising from the profunda femoris artery. (B) Selective catheterization of the involved arterial segment under fluoroscopic guidance. (C) Deployment of the covered stent across the pseudoaneurysm neck. (D) Completion angiography confirms complete exclusion of the pseudoaneurysm with preserved distal arterial flow.

Although no CT scan was performed at admission, there were neither clinical nor biological signs suggestive of a pseudoaneurysm at that time. A whole-body PET-CT performed 3 weeks prior to the trauma for oncologic staging showed no abnormality of the left PFA. The latter occurrence, following the second fall, together with the secondary displacement of the lesser trochanter, strongly supports the conclusion that this fragment ultimately played a determining role in the arterial lesion. Revision surgery was performed jointly by orthopedic and vascular teams. A covered stent was deployed after selective catheterization of the PFA (Figures 8-9), and a 5 × 25 mm ViaBahn covered stent was deployed (Figure 7).

**Figure 10:**
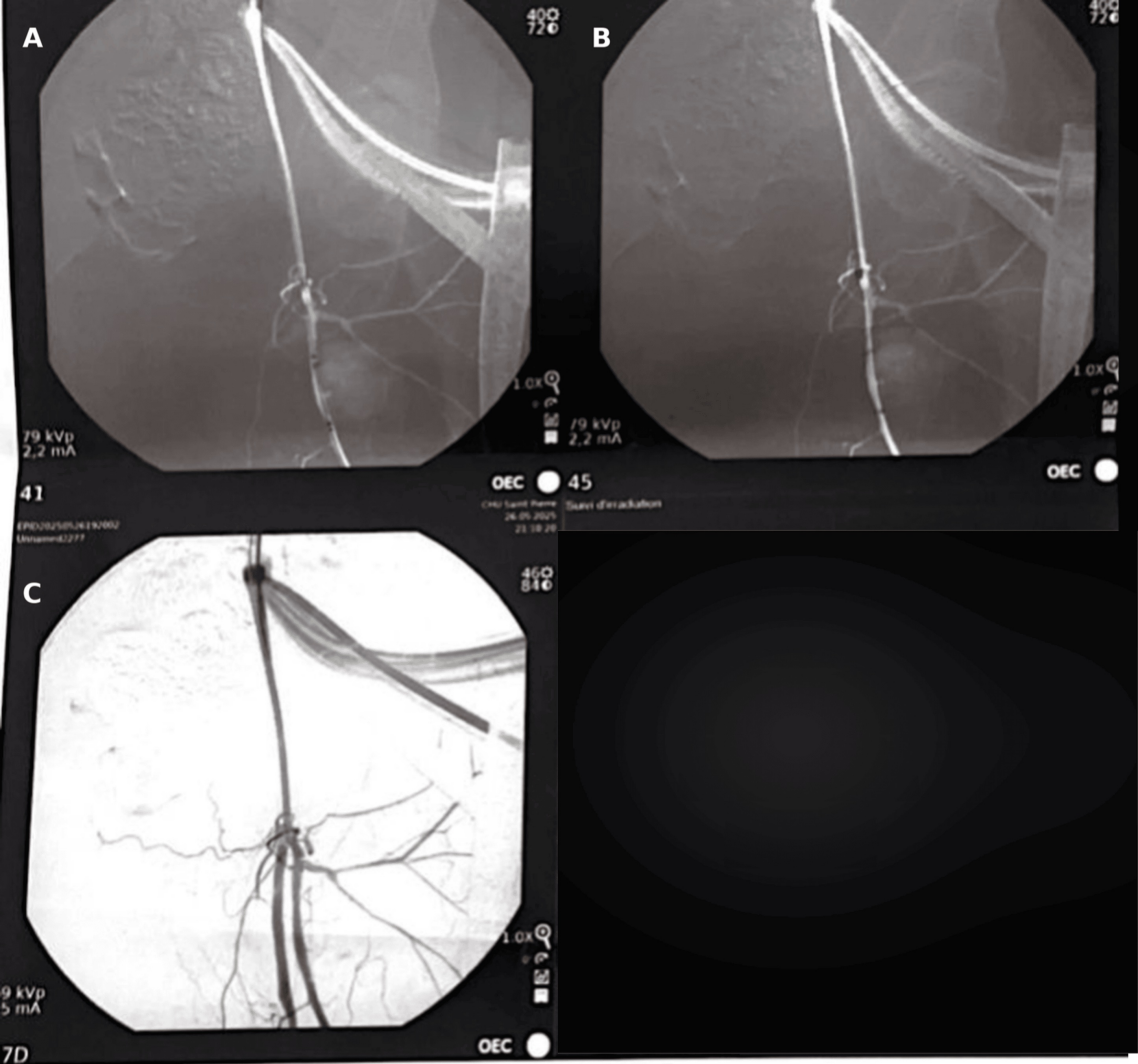
Completion angiography confirming complete exclusion of the profunda femoris artery pseudoaneurysm with preserved distal flow. (A) Anteroposterior projection demonstrating absence of contrast filling of the pseudoaneurysm after endovascular treatment. (B) Oblique projection confirming complete exclusion of the pseudoaneurysm with preserved flow in the profunda femoris artery. (C) Distal angiographic view showing maintained distal arterial perfusion without evidence of residual leak.

DSA showed complete exclusion of the pseudoaneurysm with preserved distal flow (Figure 10).

Postoperatively, wound cultures grew Staphylococcus aureus (MSSA) and Enterococcus faecalis. Intravenous vancomycin and ceftazidime were started, later switched to oral levofloxacin and rifampicin. Persistent wound drainage required debridement (DAIR), after which healing was achieved. At 2 months, radiographs confirmed fracture union. The patient was mobilizing pain-free with two crutches. Distal pulses were palpable, and there was no evidence of ischemia.

## Discussion

Hip fractures in elderly patients are frequent, but vascular complications are rare. Among them, pseudoaneurysm of the PFA is one of the most dangerous.

Multiple mechanisms of arterial injury have been described: direct iatrogenic trauma from drilling or screws, and laceration by displaced bone fragments, especially the lesser trochanter [7-9]. Our case is notable because the pseudoaneurysm was not present at initial admission but developed after a secondary fall, when the lesser trochanter fragment migrated further and came into direct contact with the artery. This emphasizes the importance of monitoring fragment displacement, as the proximity of a sharp bone fragment to the artery can be the sole cause of pseudoaneurysm.

Clinically, a pseudoaneurysm may mimic a hematoma, infection, or deep vein thrombosis. Typical signs, such as a pulsatile mass, are often absent. Persistent anemia, thigh swelling, or unusual wound drainage should raise suspicion [9].

The pathophysiology involves partial injury to the media and intima, followed by blood extravasation contained by surrounding tissues. Unlike a simple hematoma, the pseudo-aneurysmal cavity remains perfused through an arterial neck, which explains its progressive expansion. This characteristic makes Doppler ultrasound sometimes insufficient, as the appearance may be mistaken for a hematoma. In contrast, CT angiography provides a precise map of the arterial communication, sac size, and its relationship with bone structures and implants [10].

CT angiography remains the diagnostic gold standard, allowing precise assessment of the pseudoaneurysm and its relationship with fracture fragments [11,12]. Doppler ultrasound may assist, but it provides less detail.

Management depends on the clinical context. Endovascular techniques such as coil embolization or stenting are increasingly used, but open surgery is indicated when revision of fixation is required or when large hematomas complicate the situation [13,14]. In our case, a combined orthopedic and endovascular approach was employed, involving revision fixation of the displaced fragment and endovascular exclusion of the pseudoaneurysm with covered stent placement, ensuring vascular control and limb preservation.

In recent literature, most authors favor an isolated endovascular approach when bone stability is already achieved. However, in cases where a free fragment continues to injure the artery, additional orthopedic intervention is imperative to prevent recurrence. Experience shows that combining secondary fixation with vascular repair yields the best functional outcomes. Close clinical and biological monitoring (Hb, CRP, infectious status) is essential during the first weeks.

Several cases have been reported in the literature. Jain et al. described embolization for a PFA pseudoaneurysm three days after intramedullary nailing [15]. Piolanti et al. reported a similar lesion treated with stenting at 16 days [16]. Vande Voorde et al. described a late-onset pseudoaneurysm treated with coil embolization [17]. Other authors have reported silent or delayed pseudoaneurysms following intertrochanteric or proximal femoral fracture fixation, sometimes associated with fixation failure or bone erosion [18-20]. In most cases, delayed diagnosis was a challenge, highlighting the need for vigilance (Table 2).

**Table 2:** Reported cases of profunda femoris artery pseudoaneurysm following gamma nail or intramedullary fixation for subtrochanteric hip fractures

Author	Year	Fracture Type	Fixation Method	Time to Diagnosis	Diagnostic Modality	Treatment
Maheshwari et al. [3]	2004	Intertrochanteric	Internal fixation	Delayed	Angiography	Surgical repair
Agrawal et al. [4]	2020	Intertrochanteric	Internal fixation	Delayed	CT angiography	Endovascular treatment
Rajaesparan et al. [5]	2008	Intertrochanteric	Intramedullary nail	Early	Angiography	Endovascular embolization
Singh et al. [7]	2013	Intertrochanteric	Dynamic hip screw	Delayed	CT angiography	Endovascular embolization
Piolanti et al. [16]	2017	Pertrochanteric	Intramedullary nail	Delayed	CT angiography	Endovascular stenting
Vande Voorde et al. [17]	2018	Intertrochanteric	Intramedullary nailing	Late	CT angiography	Endovascular embolization
Li et al. [18]	2011	Intertrochanteric	Long intramedullary nail	Delayed	CT angiography	Coil embolization
Orapiriyakul et al. [19]	2022	Intertrochanteric	PFN + cerclage wiring	Delayed	CT angiography	Endovascular repair
Abed and Nour [20]	2017	Proximal femur	Internal fixation	Late	CT angiography	Endovascular repair

Thus, this case illustrates not only the rarity of the complication but, more importantly, the need for multidisciplinary collaboration between orthopedic surgeons, radiologists, and vascular surgeons. An institutional protocol should be considered in trauma centers to systematically evaluate any patient presenting with unexplained anemia, expanding hematoma, or persistent swelling after proximal femur surgery.

To our knowledge, this case is uncommon in two aspects: first, the causal role of the lesser trochanter fragment was clearly suggested radiologically; and second, the pseudoaneurysm developed only after a subsequent traumatic event rather than at the time of initial injury or primary surgery. This underlines the dynamic nature of fragment displacement and its potential to cause delayed vascular injury.

When identified and managed early, prognosis is excellent, with most patients achieving fracture union and full functional recovery. However, delayed recognition may result in catastrophic hemorrhage or limb loss [21,22].

Clinical message

The proximity of a displaced bone fragment, especially the lesser trochanter, to the PFA can directly cause pseudoaneurysm. Surgeons should suspect vascular injury when anemia or thigh swelling persists, particularly after secondary trauma.

## Conclusions

Pseudoaneurysm of the PFA is a rare but potentially serious complication following proximal femoral fracture fixation. This case illustrates a delayed presentation characterized by progressive anemia and thigh swelling after secondary trauma, with diagnosis confirmed by CT angiography. The spatial and temporal relationship between secondary displacement of the lesser trochanter fragment and the vascular lesion, as demonstrated on CT angiography, supports a likely mechanical contribution to arterial injury. Management with a combined orthopedic revision and endovascular stent placement resulted in satisfactory vascular control and fracture healing. Awareness of this potential complication and timely vascular imaging are essential to prevent severe outcomes.
